# Pediatric cardiomyopathy in a resource-limited setting: clinical characteristics and determinants of medium-term outcomes

**DOI:** 10.3389/fmed.2026.1800984

**Published:** 2026-07-27

**Authors:** Ghina Fakhri, Rana Zareef, Nour Younis, Nour Abdul Halim, Jad Abdul Khalek, Adham Makarem, Rand Ibrahim, Mohammed Ahmed, Fadi Fuad Bitar, Mariam Toufic Arabi

**Affiliations:** 1 Department of Pediatrics and Adolescent Medicine, American University of Beirut Medical Center, Beirut, Lebanon; 2 Division of Cardiology, Department of Pediatrics and Adolescent Medicine, American University of Beirut Medical Center, Beirut, Lebanon; 3 Faculty of Medicine, American University of Beirut, Beirut, Lebanon

**Keywords:** cardiomyopathy, dilated cardiomyopathy, hypertrophic cardiomyopathy, pediatric cardiomyopathy, restrictive cardiomyopathy

## Abstract

**Background and Aims:**

Pediatric cardiomyopathy is the leading indication for cardiac transplantation in children. However, data on its prevalence and outcomes mainly comes from developed countries, with only a scarce amount of information about its status in low- and middle-income countries.

**Purpose:**

This study aimed to characterize the clinical presentation, echocardiographic features, and outcomes of pediatric cardiomyopathy at a tertiary care center in a developing country.

**Methods:**

We conducted a retrospective review of children diagnosed with cardiomyopathy before 18 years of age at a single tertiary cardiac center over a 20-year period. Mortality at 5 years following diagnosis was assessed as a binary outcome. Univariable and multivariable logistic regression analyses were performed to identify factors associated with mortality.

**Results:**

A total of 244 children were included (mean age at diagnosis 5.9 ± 5.7 years; 57% male). Dilated cardiomyopathy was the most common phenotype, identified in 153 patients (62.7%), followed by hypertrophic cardiomyopathy in 74 (30.3%), restrictive cardiomyopathy in 10 (4.1%), and left ventricular non-compaction cardiomyopathy in 7 (2.9%). No cases of arrhythmogenic right ventricular cardiomyopathy were identified. Half of the cohort was asymptomatic at presentation and diagnosed incidentally. Presumptive myocarditis/inflammatory etiologies predominated overall and in the DCM subgroup, whereas familial/genetic etiologies predominated among patients with HCM. The 5-year mortality rate was 13.1%, with the majority of deaths occurring in children with dilated cardiomyopathy. In the overall cohort multivariable model, severely reduced left ventricular systolic function at diagnosis (adjusted OR 3.06, 95% CI 1.36–6.85) and pulmonary hypertension at diagnosis (adjusted OR 2.64, 95% CI 1.45–4.80) were the only independent predictors of mortality. Cardiomyopathy subtype, age at diagnosis, sex, and arrhythmias were not independently associated with outcome. In the In the DCM subgroup, pulmonary hypertension remained independently associated with mortality (adjusted OR 2.77, 95% CI 1.42–5.40), while severe systolic dysfunction showed a borderline association (adjusted OR 2.54, 95% CI 1.00–6.44).

**Conclusion:**

In this cohort, pediatric cardiomyopathy was characterized by a high prevalence of incidental diagnoses and a predominance of dilated phenotypes. Severe systolic dysfunction and pulmonary hypertension were the principal determinants of medium-term mortality, outweighing demographic and phenotypic factors.

## 1 Introduction

Pediatric cardiomyopathies (CM) represent a heterogeneous and complex group of myocardial disorders characterized by structural and functional abnormalities of the heart muscle that are not attributable to congenital heart disease or other identifiable structural conditions (1–3). Despite their relative rarity, pediatric cardiomyopathies are associated with substantial morbidity and mortality and remain the leading indication for heart transplantation in children older than 1 year of age (1, 2). Clinical outcomes are highly variable and reflect a complex interplay between genetic predisposition, environmental exposures, and age-dependent myocardial vulnerability (1, 2).

The reported annual incidence of pediatric cardiomyopathy is approximately 1 per 100,000 children, with marked variation according to age, sex, and population studied (2, 4, 5). Dilated cardiomyopathy (DCM) remains the most common pediatric phenotype and a major cause of pediatric heart failure, followed by hypertrophic cardiomyopathy (HCM), whereas restrictive, arrhythmogenic, and left ventricular non-compaction cardiomyopathies are less frequent (1, 2, 6). The etiologic spectrum varies considerably by phenotype and age at presentation (1, 2, 6, 7). Most pediatric HCM is related to pathogenic variants in sarcomeric genes and is more commonly recognized beyond infancy. In contrast, HCM presenting during infancy has a higher proportion of non-sarcomeric etiologies, including metabolic disorders, neuromuscular diseases, and RASopathies (7). Although these conditions represent an important subgroup of infant-onset HCM, they do not account for the majority of pediatric HCM overall. These age- and etiology-related differences are clinically relevant because syndromic and metabolic forms often present earlier, may involve extracardiac manifestations, and may have distinct disease trajectories and prognostic implications. Overlapping phenotypes may also occur, further complicating classification and risk stratification (1, 2, 6, 7).

Unlike adult cardiomyopathy, pediatric disease provides a unique window into myocardial development and dysfunction in the absence of confounding conditions such as atherosclerosis, hypertension, and diabetes (2, 6). Large registry-based studies from North America, Europe, and Australia have significantly advanced our understanding of the epidemiology, classification, and outcomes of pediatric cardiomyopathy (4, 5, 8). However, data from low- and middle-income countries remain scarce, and regional variations in disease presentation, etiology, access to advanced therapies, and outcomes are poorly defined. This gap limits global benchmarking and may hinder the development of context-specific management strategies.

The present study aims to address this knowledge gap by characterizing the epidemiology, clinical presentation, echocardiographic features, and outcomes of pediatric cardiomyopathy at a tertiary care center in a developing country. By providing contemporary regional data, this study seeks to contribute to the broader understanding of pediatric cardiomyopathy and to inform clinical practice and future research in similar healthcare settings.

## 2 Materials and methods

### 2.1 Study design and setting

This was a single-center, retrospective observational study conducted at the Children’s Heart Center of the American University of Beirut Medical Center (AUBMC), a tertiary referral center for pediatric cardiac disease in Lebanon and the surrounding region. The study protocol was reviewed and approved by the Institutional Review Board of AUBMC (IRB ID: BIO-2019-389). The requirement for informed consent was waived due to the retrospective nature of the study.

### 2.2 Study population

Medical records of all patients younger than 18 years diagnosed with cardiomyopathy between January 2000 and December 2020 were reviewed. Cardiomyopathy was classified into dilated, hypertrophic, restrictive, and left ventricular non-compaction phenotypes based on echocardiographic findings. Patients with cardiomyopathy secondary to structural congenital heart disease or incomplete clinical data were excluded. For subgroup analyses, patients were stratified according to cardiomyopathy subtype. Separate analyses were performed for DCM and HCM to account for differences in pathophysiology and outcome profiles.

### 2.3 Data collection

Demographic, clinical, electrocardiographic, echocardiographic, and outcome data were extracted from electronic medical records. Collected variables included age at diagnosis, sex, family history of cardiomyopathy or sudden cardiac death, presenting symptoms, arrhythmias, and mortality status at 5 years.

Echocardiographic parameters included left ventricular ejection fraction (EF), categorized as < 30%, 30–49%, or ≥ 50%, presence of pulmonary hypertension, left atrial dilation, mitral regurgitation severity, ventricular involvement (left ventricular, biventricular, or septal), and the presence and degree of left ventricular outflow tract (LVOT) obstruction in patients with HCM. Systolic anterior motion (SAM) of the mitral valve was also recorded in the HCM subgroup.

Etiology was assigned hierarchically to each patient based on available clinical, laboratory, imaging, and genetic data, with each patient classified under a single primary etiology (presumptive myocarditis/inflammatory, familial/genetic, metabolic, idiopathic, chemotherapy-related or undetermined).

Etiological classification of myocarditis was primarily based on clinical presentation, laboratory findings, and echocardiography, as cardiac MRI and endomyocardial biopsy were not routinely available.

Pulmonary hypertension (PH) at diagnosis was abstracted from the contemporaneous echocardiography report. Echocardiographic evidence suggestive of pulmonary hypertension was considered present when the contemporaneous echocardiography report documented a tricuspid regurgitation jet-derived estimated right ventricular systolic pressure greater than 35 mmHg, after accounting for estimated right atrial pressure and in the absence of right ventricular outflow tract obstruction, and/or supportive findings such as interventricular septal flattening, shortened pulmonary artery acceleration time, or pulmonary regurgitation jet-derived pressure estimation. Because right-heart catheterization was not routinely performed, this variable was interpreted as a noninvasive marker of elevated pulmonary pressure rather than catheter-confirmed pulmonary hypertension (9).

### 2.4 Outcome measures

The primary outcome was all-cause mortality within 5 years of cardiomyopathy diagnosis. Survival status was determined from medical records and follow-up documentation. Secondary outcomes included associations between clinical and echocardiographic variables and mortality within cardiomyopathy subgroups. Severity of cardiomyopathy was defined using objective echocardiographic criteria at the time of diagnosis. In patients with dilated, restrictive, or left ventricular non-compaction cardiomyopathy, severity was classified based on left ventricular EF. LVEF at diagnosis was categorized according to baseline echocardiographic LVEF as < 30%, 30%–49%, and ≥ 50%. These categories were selected to distinguish severe systolic dysfunction from intermediate dysfunction and relatively preserved systolic function, while avoiding over-stratification in a modest-sized retrospective cohort. This approach is broadly aligned with recent pediatric heart failure guidance, in which severe systemic LV dysfunction is defined at EF ≤ 30%, moderate dysfunction at 31%–40%, mild dysfunction at 41%–51%, and normal systolic function at ≥ 52% (10).

In patients with HCM, severity was determined according to the presence and degree of LVOT obstruction, ventricular hypertrophy, and systolic function, with severe disease defined by significant LVOT obstruction ( ≥ 50 mmHg), systolic dysfunction, or biventricular involvement. When multiple features were present, patients were classified according to the highest severity category. Importantly, this classification was used for descriptive purposes within the study and does not reflect contemporary risk stratification models for hypertrophic cardiomyopathy.

### 2.5 Statistical analysis

Descriptive statistics were used to summarize patient characteristics. Continuous variables were reported as mean ± standard deviation or median (interquartile range), as appropriate, while categorical variables were presented as frequencies and percentages. Group comparisons were performed using the Mann–Whitney U test for non-normally distributed continuous variables and the chi-square test or Fisher’s exact test for categorical variables, as appropriate.

The primary outcome was all-cause mortality within 5 years of cardiomyopathy diagnosis (binary outcome). Due to variability in follow-up duration and incomplete time-to-event data inherent to the retrospective design, formal survival analysis using Kaplan–Meier or Cox proportional hazards models was not feasible. Therefore, a fixed 5-year mortality endpoint was selected to allow consistent outcome assessment across the cohort. To identify predictors of 5-year mortality in the overall cohort, univariable logistic regression was first performed for candidate demographic, clinical, electrocardiographic, echocardiographic, and etiological variables. Variables with clinical relevance and/or a univariable *p*-value < 0.1 were entered into a multivariable logistic regression model to determine independent predictors of 5-year mortality in the full cohort, with effect estimates reported as odds ratios (ORs) and 95% confidence intervals (CIs).

Given heterogeneity across phenotypes and the predominance of DCM, a prespecified subgroup analysis was conducted to identify independent predictors of 5-year mortality among patients with DCM.

For the HCM subgroup, multivariable regression was not performed due to the limited number of mortality events and risk of model overfitting. Instead, predictors and group differences within HCM were assessed using univariable analyses and exact statistical tests (Fisher’s exact test) where appropriate.

Statistical significance was defined as a two-sided *p*-value < 0.05. All analyses were performed using IBM SPSS Statistics (version 20.0; IBM Corp., Armonk, NY, USA).

## 3 Results

### 3.1 Baseline demographic and clinical characteristics

A total of 244 medical records were reviewed, corresponding to children newly diagnosed with cardiomyopathy before the age of 18 years at our tertiary cardiac center. The mean age at diagnosis was 5.9 ± 5.7 years, with a male predominance (57%). Ninety patients (36.9%) were diagnosed during infancy, and more than half of the cohort (57%) received the diagnosis at or before 5 years of age. The study population was predominantly Lebanese, with only 3.3% of patients being non-Lebanese. Notably, nearly one-fifth of patients had a positive family history of childhood cardiac disease, and approximately 6% had siblings who died before the age of 10 years due to cardiac causes. Detailed demographic characteristics are summarized in Table 1.

**TABLE 1:** Baseline demographic and medical characteristics of the population.

Variable	Total (*N* = 244)
Age at diagnosis, years (mean ± SD)	5.9 ± 5.7
Male sex, *n* (%)	140 (57.4)
Nationality, *n* (%)	
Lebanese	236 (96.7)
Non-Lebanese	8 (3.3)
Family history of childhood heart disease, *n* (%)	46 (18.9)
Family history of cardiac-related death < 10 years, *n* (%)	15 (6.1)
Age group at diagnosis, *n* (%)	
Infancy ( ≤ 1 year)	90 (36.9)
≤ 5 years	139 (57.0)
Symptoms at presentation, *n* (%)	
Asymptomatic (incidentally detected)	123 (50.4)
Respiratory symptoms	92 (37.7)
Cardiac symptoms	29 (11.9)
Type of cardiomyopathy, *n* (%)	
Dilated cardiomyopathy	153 (62.7)
Hypertrophic cardiomyopathy	74 (30.3)
Restrictive cardiomyopathy	10 (4.1)
Left ventricular non-compaction	7 (2.9)
*Severity of cardiomyopathy at diagnosis, n (%)* *	
Mild	108 (44.3)
Moderate	87 (35.7)
Severe	49 (20.1)

*Severity was defined using echocardiographic criteria at diagnosis. mild, moderate, and severe categories were based on left ventricular ejection fraction for non-hypertrophic cardiomyopathies and on degree of obstruction and ventricular involvement for hypertrophic cardiomyopathy.

Respiratory symptoms were the most common presenting feature, reported in 37.7% of patients. On physical examination, signs of heart failure including tachycardia, crackles, hepatomegaly, pathologic murmurs and poor perfusion were observed in minority of patients, 9.8–16.8% of cases. A substantial proportion of patients (46.3%) had no significant abnormal findings. Half of the cohort was asymptomatic at presentation and diagnosed incidentally; in these cases, echocardiography was performed for screening due to a positive family history of cardiomyopathy, the presence of metabolic or neuromuscular disorders requiring routine cardiac surveillance, or the incidental detection of a cardiac murmur. When stratified by era of presentation, incidental diagnosis of cardiomyopathy was observed in 28/93 (30%) before 2010 compared with 95/151 (62.9%) from 2010 onward.

DCM was the most prevalent subtype, accounting for 62.7% of cases, followed by HCM (30.3%). Restrictive and left ventricular non-compaction cardiomyopathies were uncommon, representing 4.1% and 2.9% of the cohort, respectively. Interestingly, no cases of arrhythmogenic right ventricular cardiomyopathy were identified in this cohort. Nearly half of the patients (44.3%) presented with mild cardiomyopathy, whereas severe disease was observed in 20% of cases. Across all subtypes, presumably viral/myocarditis or inflammatory-related processes (28.3%) and familial or hereditary conditions (20.9%) were the most frequently identified etiologies.

Echocardiographic evidence suggestive of pulmonary hypertension at diagnosis was present in 40 of 244 patients (16.4%), including 31 (12.7%) with mild, 7 (2.9%) with moderate, and 2 (0.8%) with severe PH.

With respect to treatment approach, among survivors (*n* = 212), 151 (71.2%) received medical therapy, 32 (15.1%) underwent surgical intervention, and 29 (13.7%) had no recorded intervention. Advanced heart failure therapies were rarely utilized in this cohort. One patient underwent heart transplantation and was alive at follow-up. One additional patient underwent LVAD implantation outside our institution and died shortly thereafter.

### 3.2 Electrocardiographic and echocardiographic findings

All patients underwent electrocardiographic (ECG) evaluation at diagnosis as part of their initial assessment. ECG findings were normal in 68 patients. Sinus tachycardia was the most common abnormality (*n* = 106), followed by voltage criteria for left ventricular hypertrophy (*n* = 32) and left bundle branch block (*n* = 8). Ventricular ectopy was identified in 10 patients (4.1%), supraventricular tachycardia in 8 (3.3%), and premature atrial contractions in 25 (10.2%). Atrial rhythm was noted in 8 patients (3.3%), and complete or incomplete right bundle branch block in 18 patients (7.35%).

**FIGURE 1:**
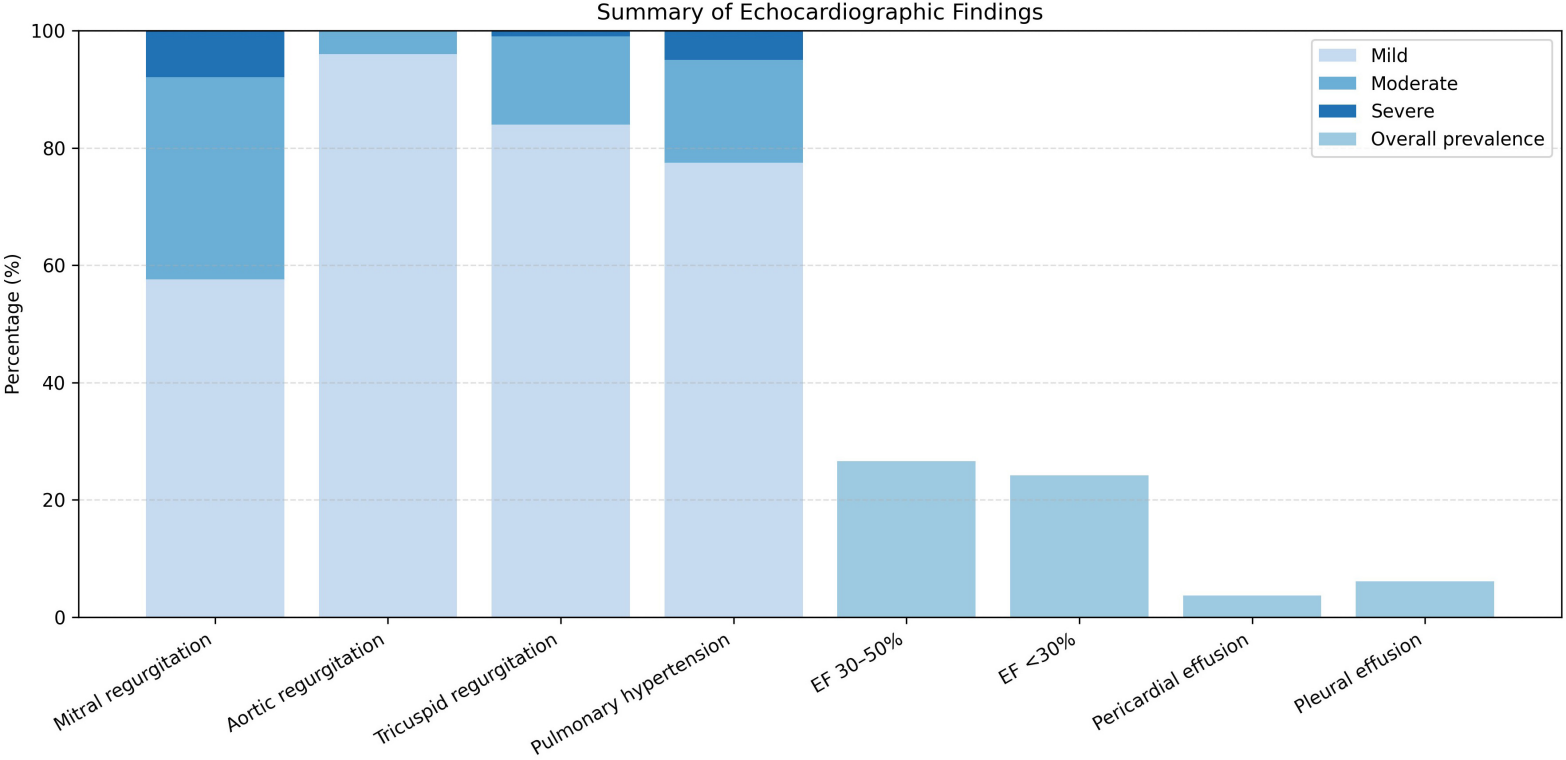
Summary of echocardiographic findings in the pediatric cardiomyopathy cohort. Stacked bars represent the severity distribution (mild, moderate, severe) of valvular regurgitation and pulmonary hypertension among affected patients, while single bars indicate overall prevalence of left ventricular systolic dysfunction, pericardial effusion, and pleural effusion.

Echocardiographic findings are summarized in Figure 1. Approximately one-quarter of patients demonstrated severely depressed left ventricular systolic function, with an EF < 30%. Mitral regurgitation was present in 72.5% of cases, including moderate to severe regurgitation in 11.3%. Aortic regurgitation was observed in 10.2% of patients, with no cases of severe regurgitation. Pulmonary hypertension was identified in 16.4% of the cohort; among these, 7 patients (17.5%) had an estimated pulmonary artery systolic pressure of 41–55 mmHg, and 2 patients (5%) exceeded 55 mmHg. Biventricular involvement was present in 8.6% of patients overall and was uncommon among those with DCM (3.9%).

### 3.3 Predictors of mortality

The overall 5-year survival rate was 86.9%, with 13.1% of patients not surviving beyond 5 years following diagnosis. Of the patients who died, 75% had DCM, and nearly half presented with severe disease at diagnosis. Univariable logistic regression (Table 2) was performed to identify factors associated with 5-year mortality. Severely reduced left ventricular systolic function at presentation, defined as an ejection fraction < 30%, was significantly associated with increased mortality (OR 3.93, 95% CI 1.82–8.49, *p* < 0.001). Similarly, the presence of pulmonary hypertension at diagnosis was a strong predictor of adverse outcome, conferring a threefold increase in the odds of death within 5 years (OR 3.05, 95% CI 1.73–5.39, *p* < 0.001).

**TABLE 2:** Univariable and multivariate logistic regression analysis of predictors of 5-year mortality.

Univariate analysis	OR	95% CI	*p*-value
Ejection fraction < 30%	3.93	1.82 – 8.49	< 0.001*
Pulmonary hypertension	3.05	1.73 – 5.39	< 0.001*
Dilated cardiomyopathy	1.93	0.83 – 4.50	0.13
Age at diagnosis(non-infant vs infant)	0.62	0.29 – 1.31	0.21
Female sex	1.23	0.59 – 2.60	0.58
Arrhythmia at presentation	1.30	0.62 – 2.75	0.48
**Multivariate analysis**	**Adjusted OR**	**95% CI**	***p*-value**
Ejection fraction < 30%	3.06	1.36 – 6.85	0.007*
Pulmonary hypertension	2.64	1.45 – 4.80	0.001*

CI, Confidence Interval; OR, Odds Ratio; EF, ejection fraction.

*Statistically significant.

DCM showed a non-significant trend toward higher mortality compared with non-dilated forms (OR 1.93, 95% CI 0.83–4.50, *p* = 0.13). Age at diagnosis (infant versus non-infant), sex, and the presence of arrhythmias at presentation were not significantly associated with 5-year mortality.

Multivariable logistic regression analysis (Table 2) was performed to identify independent predictors of 5-year mortality. After adjustment, both severely reduced left ventricular systolic function and pulmonary hypertension at presentation remained independently associated with mortality. An ejection fraction < 30% was associated with a threefold increase in the odds of death within 5 years (adjusted OR 3.06, 95% CI 1.36–6.85, *p* = 0.007). Similarly, the presence of pulmonary hypertension conferred a significantly higher risk of mortality (adjusted OR 2.64, 95% CI 1.45–4.80, *p* = 0.001).

The overall model explained approximately 16% of the variance in 5-year mortality (Nagelkerke R^2^ = 0.158). Other clinical variables, including age at diagnosis, sex, cardiomyopathy subtype, and arrhythmias, were not independently associated with mortality and were therefore not retained in the final model.

### 3.4 DCM

Among the 153 children with dilated cardiomyopathy, with their corresponding characteristics summarized in Table 3. The mean age at diagnosis was 5.7 ± 5.7 years (median 4 years), with a male predominance (57.5%). At presentation, over one-third of patients (35.9%) had severe left ventricular systolic dysfunction with an EF < 30%, while an additional 36.6% had moderately reduced systolic function (EF 30–49%). Pulmonary hypertension was identified in 22.9% of patients.

**TABLE 3:** Clinical, echocardiographic, and etiological characteristics of children with dilated cardiomyopathy.

Parameter	*N* (%); Mean ± SD
Age at diagnosis, years	5.7 ± 5.7 (median 4)
Infant diagnosis ( ≤ 1 year)	35 (22.9%)
Male sex	88 (57.5%)
Mortality within 5 years form diagnosis	24 (15.7%)
Echocardiographic characteristics	
Left ventricular ejection fraction < 30%	55 (35.9%)
Left ventricular ejection fraction 30–49%	56 (36.6%)
Preserved systolic function (EF ≥ 50%)	42 (27.5%)
Pulmonary hypertension (any severity)	35 (22.9%)
Affected myocardial region	
Left ventricle only	141 (92.2%)
Biventricular involvement	6 (3.9%)
Others	6 (3.9%)
Mitral regurgitation ≥ moderate	57 (37.3%)
Arrhythmias	69 (45.1%)
Etiology	
presumptive myocarditis/inflammatory	68 (44.4%)
Metabolic	34 (22.2%)
Idiopathic	30 (19.6%)
Familial / genetic	8 (5.2%)
Chemotherapy-related	7 (4.6%)
Undetermined	6 (3.9%)

SD, standard deviation; EF, ejection fraction.

In the DCM subgroup, severe disease was defined as LVEF < 30% at diagnosis. Based on this definition, 55 patients (35.9%) had severe LV systolic dysfunction at presentation, 56 (36.6%) had intermediate systolic dysfunction, and 42 (27.5%) had preserved systolic function. Echocardiographic evidence suggestive of pulmonary hypertension was present in 35 patients (22.9%), and moderate or greater mitral regurgitation was present in 57 patients (37.3%).

Detailed treatment data were not uniformly available for the DCM subgroup because of the retrospective design and the long study period. In the overall cohort, treatment was recorded broadly as medical therapy, surgical intervention, or no documented intervention. However, medication classes, respiratory support requirements, and specific surgical or device-based interventions were not consistently captured and therefore could not be analyzed separately. Advanced heart failure therapies were rarely used; one patient underwent heart transplantation and was alive at follow-up, while one additional patient underwent LVAD implantation outside our institution and died shortly thereafter.

Similarly, genetic testing was not systematically available across the 20-year study period. Although familial or genetic etiology was assigned when supported by available clinical, family-history, or genetic information, molecular testing modality and results were not consistently documented. Therefore, the proportion of genetically confirmed cardiomyopathy could not be reliably reported, and genetic etiologies were likely underestimated.

Echocardiographic involvement was predominantly confined to the left ventricle (92.2%), with biventricular involvement observed in a minority of cases (3.9%). Moderate or greater mitral regurgitation was present in 37.3% of patients, and arrhythmias were documented in 45.1% at diagnosis.

The most common etiologies were the presumptive myocarditis/inflammatory-related (44.4%) and metabolic disorders (22.2%), followed by idiopathic (19.6%), familial (5.2%), and chemotherapy-related cardiomyopathy (4.6%). At 5-year follow-up, 24 patients (15.7%) had died.

**FIGURE 2:**
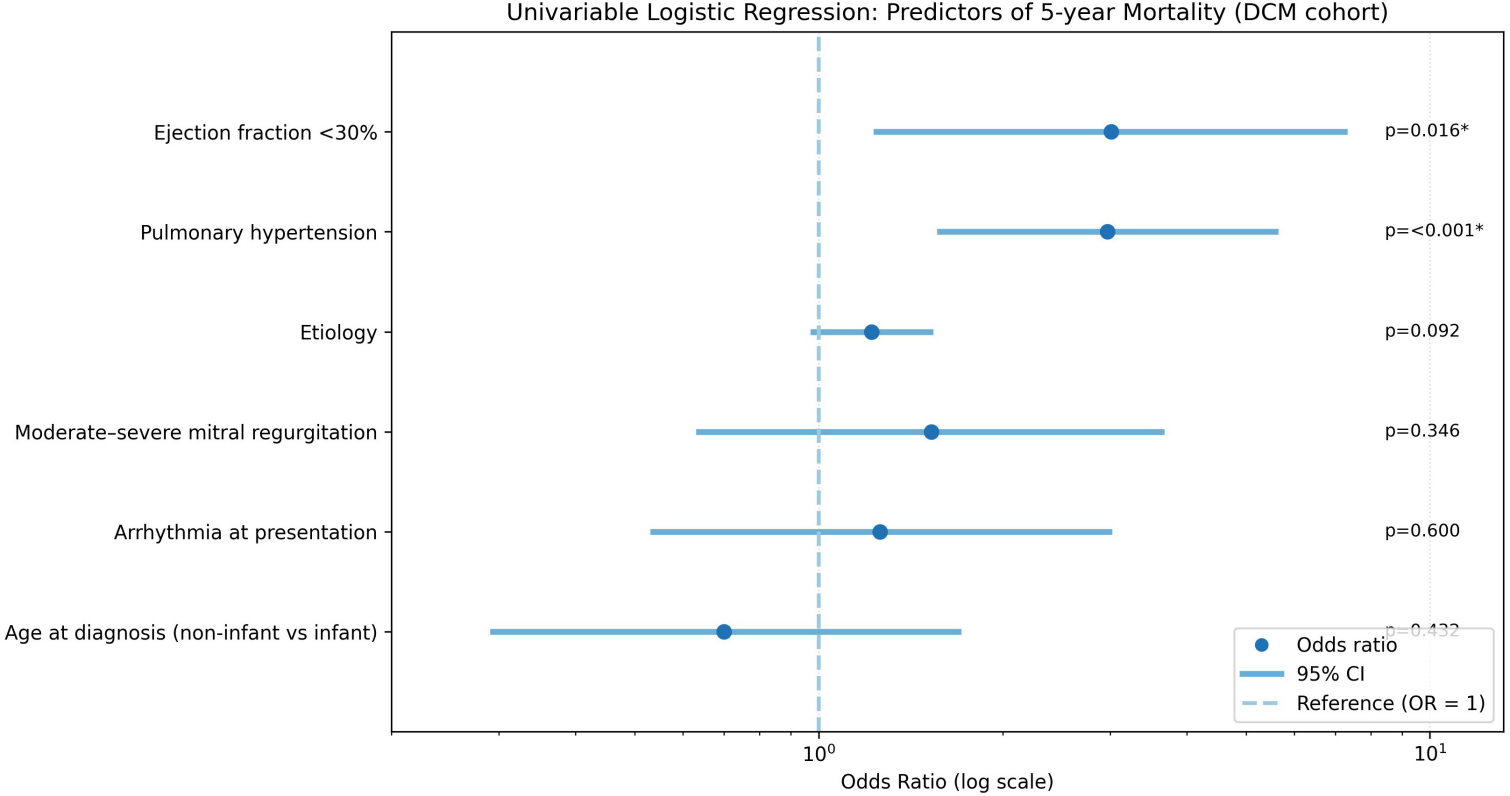
Forest plot of univariable logistic regression analyses showing predictors of 5-year mortality in children with dilated cardiomyopathy. Odds ratios (ORs) are displayed on a logarithmic scale with corresponding 95% confidence intervals (horizontal bars). The dashed vertical line indicates the null value (OR = 1). Predictors with ORs > 1 are associated with increased odds of mortality, whereas ORs < 1 indicate reduced odds. *P*-values for each predictor are shown adjacent to the estimates. *Signifies statistically significant.

Univariable logistic regression analysis was performed within the dilated cardiomyopathy subgroup (Figure 2) to identify factors associated with 5-year mortality. Severe left ventricular systolic dysfunction at presentation, defined as an ejection fraction < 30%, was significantly associated with increased mortality (OR 3.01, 95% CI 1.23–7.33, *p* = 0.016). Similarly, the presence of pulmonary hypertension at diagnosis was a strong predictor of adverse outcome, conferring nearly a threefold increase in the odds of death (OR 2.97, 95% CI 1.56–5.65, *p* < 0.001). In contrast, age at diagnosis, sex, underlying etiology, arrhythmias, and the presence of moderate to severe mitral regurgitation were not significantly associated with 5-year mortality in this subgroup.

Multivariable logistic regression analysis was performed within the DCM subgroup to identify independent predictors of 5-year mortality. After adjustment, pulmonary hypertension at presentation remained a strong independent predictor of mortality (adjusted OR 2.77, 95% CI 1.42–5.40, *p* = 0.003). Severe left ventricular systolic dysfunction, defined as an ejection fraction < 30%, showed a borderline association with 5-year mortality after adjustment (adjusted OR 2.54, 95% CI 1.00–6.44, *p* = 0.050).

### 3.5 HCM

Among the 74 children diagnosed with hypertrophic cardiomyopathy, the mean age at diagnosis was 6.6 ± 6.2 years, with 40.6% diagnosed during infancy. A male predominance was observed (59.4%), and nearly one-quarter of patients had a positive family history of cardiomyopathy. 5-year mortality in the HCM subgroup was 6.8%. The clinical and echocardiographic characteristics of this group are summarized in Table 4.

**TABLE 4:** Clinical, echocardiographic, and etiologic characteristics of children with hypertrophic cardiomyopathy.

Parameter	*N* (%); Mean ± SD
Age at diagnosis, years	6.6 ± 6.2
Infant diagnosis (≤ 1 year)	30 (40.6%)
Male sex	44 (59.4%)
Positive family history of cardiomyopathy	17 (23%)
Mortality within 5 years form diagnosis	5 (6.8%)
LVOT obstruction	
None	32 (43.2%)
Any LVOT obstruction	42 (56.8%)
Mild	26 (35.1%)
Moderate	10 (13.5%)
Severe	6 (8.1%)
Systolic anterior motion (SAM)	15 (20.3%)
Affected myocardial region	
Biventricular involvement	11 (14.9%)
Interventricular septum predominant	17 (22.9%)
Mitral regurgitation ≥ moderate	12 (16.2%)
Arrhythmias	31 (41.8%)
Etiology	
Familial / genetic	42 (56.8%)
Metabolic	7 (9.5%)
Idiopathic / Not available	25 (33.7%)

SD; standard deviation; EF, ejection fraction; LVOT, left ventricular outflow tract.

Echocardiographic evaluation demonstrated preserved left ventricular systolic function in the vast majority of patients, with no cases exhibiting an ejection fraction below 30% and only 8.7% showing mildly reduced systolic function. LVOT obstruction was present in 56.8% of patients, including moderate to severe obstruction in 21.6%, and systolic anterior motion of the mitral valve was identified in 20.3%. Biventricular hypertrophy was observed in 14.9% of cases, while arrhythmias were documented in 41.8% of the cohort.

Familial or genetic etiologies accounted for 56.8% of HCM cases, while metabolic etiologies accounted for 9.5%. Etiology was idiopathic or unavailable in 33.7% of patients.

5-year mortality was low in the HCM cohort. Mortality occurred in 4 of 32 patients (12.5%) without LVOT obstruction and in 1 of 42 patients (2.4%) with LVOT obstruction. This difference did not reach statistical significance (Fisher’s exact test, *p* = 0.159). Age at diagnosis was comparable between patients with obstructive and non-obstructive HCM (*p* = 0.80).

## 4 Discussion

This study presents a comprehensive 20-year experience of pediatric cardiomyopathy at a tertiary care center in a developing country in the Middle East, encompassing 244 children diagnosed before the age of 18 years. Historically, most knowledge regarding pediatric cardiomyopathy has been derived from large registries and cohorts in high-income countries, particularly the Pediatric Cardiomyopathy Registry established in the United States in 1994, as well as studies from Europe, Australia, and North America (5). In contrast, data from low- and middle-income countries remain sparse, with only limited reports emerging from regions such as North Africa in recent years (11–13). The absence of national registries, limited access to advanced therapies, and substantial financial barriers in these settings have contributed to a significant gap in the literature. Our study helps address this gap by providing long-term, region-specific data on epidemiology, clinical characteristics, and outcomes.

The mean age at diagnosis in our cohort was 5.9 years, with a broad spectrum of clinical presentation ranging from asymptomatic disease to advanced heart failure. Although published incidence rates suggest that cardiomyopathy is most frequent during infancy, the true national incidence in our setting remains unknown due to the absence of a population-based registry and the likelihood of underdiagnosis related to delayed healthcare access.

A notable finding in our cohort was the high proportion of incidentally detected cardiomyopathy. Rather than suggesting an unusually benign disease spectrum, this likely reflects a combination of context-specific mechanisms of case ascertainment. First, structured surveillance of children with metabolic or neuromuscular disorders can increase detection of preclinical cardiomyopathy before overt heart failure develops. Second, family screening in the presence of cardiomyopathy or sudden death history may lead to diagnosis in asymptomatic relatives earlier in the disease course. Third, as a tertiary referral center, our institution may receive a disproportionate share of screen-detected or “work-up” cases, introducing referral and selection effects that enrich the cohort for earlier-stage disease.

Genetic and familial factors play a central role in pediatric cardiomyopathy, as reflected by the substantial proportion of patients in our cohort with a positive family history of childhood cardiac disease (approximately 20%) and a history of cardiac-related death in siblings younger than 10 years (6%). These findings are consistent with the known genetic heterogeneity of cardiomyopathy, in which mutations in a single gene may give rise to different phenotypes, while similar phenotypes may arise from diverse genetic defects (14).

Importantly, the low proportion of familial/genetic etiologies in DCM likely reflects limited access to systematic genetic testing rather than true disease distribution. In settings where comprehensive genetic evaluation is available, pathogenic variants are identified in a substantial proportion of pediatric DCM cases. Therefore, underdiagnosis of genetic cardiomyopathy is likely in our cohort.

In line with international data, DCM was the most prevalent subtype in our population, accounting for nearly two-thirds of cases, followed by HCM (15). The lower proportion of severe DCM at presentation compared with some international reports should be interpreted cautiously and may reflect referral and transport patterns rather than a truly milder disease spectrum. In Lebanon, children presenting to smaller or peripheral centers with fulminant heart failure may not always reach a tertiary pediatric cardiac center because of delayed recognition, limited pediatric cardiac transport systems, financial barriers, or early death before referral. Conversely, our institution may receive a relatively higher proportion of children referred for family screening, metabolic or neuromuscular surveillance, or outpatient evaluation of incidental murmurs, thereby enriching the cohort for earlier-stage or screen-detected disease. Therefore, the observed severity distribution likely reflects both biological disease characteristics and system-level case ascertainment effects.

Restrictive and left ventricular non-compaction cardiomyopathies were rare in our cohort, mirroring their low reported incidence worldwide. Although these subtypes represented a small proportion of cases, their clinical significance remains substantial given their association with poor outcomes and limited therapeutic options.

The overall 5-year mortality rate in our cohort was 13.1%, with the majority of deaths occurring among patients with DCM. Outcomes in pediatric cardiomyopathy are known to be highly variable, ranging from complete recovery to progression to end-stage heart failure requiring transplantation. The relatively high mortality observed in our population likely reflects systemic factors, most notably the absence of advanced heart failure therapies such as ventricular assist devices and cardiac transplantation, as well as delayed presentations related to financial and healthcare access barriers. These constraints are characteristic of many low- and middle-income countries and substantially influence outcomes. Besides, because many international studies report death/transplant rather than death alone, and because access to transplantation varies widely, direct numerical comparisons should be made cautiously.

When compared with data from high-income countries, mortality rates in our cohort appear broadly comparable but must be interpreted within important contextual differences. In the Pediatric Cardiomyopathy Registry (PCMR) and other large North American cohorts, reported 5-year rates of death or transplantation range between approximately 20–40% for dilated cardiomyopathy, with transplantation accounting for a substantial proportion of adverse outcomes.

In our setting, advanced heart failure therapies were rarely utilized in this cohort, with only one patient undergoing heart transplantation and one additional patient receiving LVAD support outside our institution. Access to such therapies was limited and inconsistent across the study period, often depending on referral pathways, availability outside the country, and family financial capacity. These system-level limitations provide important context for our findings: in an environment where escalation to transplant/VAD and advanced phenotyping may be restricted, early and widely obtainable echocardiographic markers, particularly severe LV systolic dysfunction and pulmonary hypertension, may dominate risk stratification and observed outcomes.

In our cohort, echocardiographic evidence suggestive of PH at diagnosis was present in 16.4% of patients and was independently associated with 5-year mortality. This likely reflects more advanced disease at presentation, as PH in pediatric cardiomyopathy may result from chronically elevated left-sided filling pressures, secondary pulmonary vascular changes, and more severe ventricular dysfunction. Accordingly, PH at diagnosis may serve as an important noninvasive marker of hemodynamic compromise and higher-risk presentation. Because PH was defined echocardiographically rather than invasively, it should be interpreted as a clinically relevant severity marker rather than a definitive hemodynamic diagnosis.

As such, due to these important limitations, outcomes in our cohort likely reflect a combination of true disease severity and system-level constraints, rather than direct comparability with composite endpoints from high-resource settings. These disparities underscore the importance of contextualizing outcome data and highlight the need for region-specific risk stratification and management strategies.

Importantly, severely reduced left ventricular systolic function and pulmonary hypertension at diagnosis emerged as the only independent predictors of 5-year mortality in our cohort. After multivariable adjustment, an ejection fraction below 30% conferred a threefold increased risk of death, while the presence of pulmonary hypertension was associated with an approximately 2.6-fold higher risk.

While the prognostic significance of severe systolic dysfunction and pulmonary hypertension is well established in pediatric cardiomyopathy, the contribution of this study lies in confirming the relevance of these markers within a resource-limited healthcare setting. In such environments, where access to advanced diagnostics and therapies is variable, the ability of widely available echocardiographic parameters to reliably stratify risk is of particular clinical importance.

Although our sample size is smaller than large multicenter registries, it represents one of the largest single-center cohorts from the Middle East with long-term outcome data. Given the scarcity of regional data, such studies are essential to contextualize global evidence and inform locally applicable clinical decision-making.

Notably, cardiomyopathy subtype, age at diagnosis, sex, and arrhythmias were not independently associated with mortality after adjustment. In this cohort, cardiomyopathy phenotype did not retain an independent association with mortality after adjustment, whereas markers of hemodynamic severity remained independently associated with outcome. Similarly, while arrhythmias and infant-onset disease have been linked to poor outcomes in other cohorts, their impact in our population appears secondary to markers of advanced disease at presentation.

Additionally, the retrospective design limited the availability and uniform application of advanced diagnostic modalities such as cardiac magnetic resonance imaging and comprehensive genetic testing. These limitations, largely driven by financial and resource constraints, may have resulted in under-characterization of underlying etiologies and incomplete phenotypic classification in a subset of patients. Additionally, the lack of detailed time-to-event data precluded formal survival analysis, necessitating the use of a fixed 5-year mortality outcome. While this approach reduces temporal resolution, it allows for consistent outcome assessment across a heterogeneous cohort. Loss to follow-up, largely driven by socioeconomic factors, further limited long-term outcome assessment.

Although our institution follows a protocol-based approach to pediatric heart failure management, tailored to the underlying cardiomyopathy subtype and hemodynamic status, granular treatment data were not consistently available across this retrospective cohort. In general, management at our center includes standard pediatric heart failure therapies such as diuretics for volume overload, ACE inhibitors for afterload reduction when appropriate, beta-blockers in selected patients with systolic dysfunction, digoxin in selected cases, and escalation to intensive care support or surgical intervention when clinically indicated (16). However, detailed data on individual medication classes, antiarrhythmic therapy, ICD implantation, and specific surgical procedures were not uniformly captured. Accordingly, treatment heterogeneity could not be fully accounted for and may have confounded the observed associations with outcomes.

Although this was a single-center study, our institution serves as a major tertiary referral center for pediatric cardiac disease in Lebanon and the surrounding region. As such, the cohort likely reflects a wide spectrum of disease severity and referral patterns encountered in similar resource-limited settings. Nonetheless, caution is warranted when extrapolating these findings to other healthcare systems with different infrastructures and access to advanced therapies.

Despite these limitations, our study provides valuable insight into pediatric cardiomyopathy in a resource-limited setting and highlights the central role of disease severity at presentation in determining outcomes. These findings underscore the importance of early diagnosis, systematic echocardiographic risk stratification, and targeted follow-up strategies. Multicenter studies and regional registries from low- and middle-income countries are urgently needed to better define disease burden, improve prognostication, and guide context-appropriate management strategies for this rare but impactful condition.

## Data Availability

The data analyzed in this study is subject to the following licenses/restrictions: The dataset supporting the conclusions of this article is available from the corresponding author upon reasonable request. Requests to access these datasets should be directed to ma81@aub.edu.lb.

## References

[1] Lipshultz SE Law YM Asante-Korang A Austin ED Dipchand AI Everitt MD et al. Cardiomyopathy in children: classification and diagnosis: a scientific statement from the American heart association. *Circulation.* (2019) 140:e9–68. 10.1161/CIR.0000000000000682 31132865

[2] Rath A Weintraub R. Overview of cardiomyopathies in childhood. *Front Pediatr.* (2021) 9:708732. 10.3389/fped.2021.708732 34368032 PMC8342800

[3] Arbelo E Protonotarios A Gimeno JR Arbustini E Barriales-Villa R Basso C et al. 2023 ESC Guidelines for the management of cardiomyopathies. *Eur Heart J.* (2023) 44:3503–626. 10.1093/eurheartj/ehad194 37622657

[4] Nugent AW Daubeney PEF Chondros P Carlin JB Cheung M Wilkinson LC et al. The epidemiology of childhood cardiomyopathy in Australia. *N Engl J Med.* (2003) 348:1639–46. 10.1056/NEJMoa021737 12711738

[5] Lipshultz SE Sleeper LA Towbin JA Lowe AM Orav EJ Cox GF et al. The incidence of pediatric cardiomyopathy in two regions of the United States. *N Engl J Med.* (2003) 348:1647–55. 10.1056/NEJMoa021715 12711739

[6] Lee TM Hsu DT Kantor P Towbin JA Ware SM Colan SD et al. Pediatric cardiomyopathies. *Circ Res.* (2017) 121:855–73. 10.1161/CIRCRESAHA.116.309386 28912187 PMC5657298

[7] Townsend M Jeewa A Khoury M Cunningham C George K Conway J. Unique aspects of hypertrophic cardiomyopathy in children. *Can J Cardiol.* (2024) 40:907–20. 10.1016/j.cjca.2024.01.013 38244986

[8] Arola A Jokinen E Ruuskanen O Saraste M Pesonen E Kuusela AL et al. Epidemiology of idiopathic cardiomyopathies in children and adolescents: a nationwide study in Finland. *Am J Epidemiol.* (1997) 146:385–93. 10.1093/oxfordjournals.aje.a009291 9290498

[9] Jone PN Ivy DD. Echocardiography in pediatric pulmonary hypertension. *Front Pediatr.* (2014) 2:124. 10.3389/fped.2014.00124 25429362 PMC4228850

[10] Amdani S Conway J George K Martinez HR Asante-Korang A Goldberg CS et al. Evaluation and management of chronic heart failure in children and adolescents with congenital heart disease: a scientific statement from the American heart association. *Circulation.* (2024) 150:e33–50. 10.1161/CIR.0000000000001245 38808502

[11] Bakeet MAE Mohamed MM Allam AA Gamal R. Childhood cardiomyopathies: a study in a tertiary care hospital in Upper Egypt. *Electron Physician.* (2016) 8:3164–9. 10.19082/3164 28070248 PMC5217807

[12] Elmasry OA Kamel TB El-Feki NF. Pediatric cardiomyopathies over the last decade: a retrospective observational epidemiology study in a tertiary institute, Egypt. *J Egypt Public Health Assoc.* (2011) 86:63–7. 10.1097/01.EPX.0000399140.68151.6a 21844761

[13] Bagkaki A Parthenakis F Chlouverakis G Anastasakis A Papagiannis I Galanakis E et al. Epidemiology of pediatric cardiomyopathy in a mediterranean population. *Children.* (2024) 11:732. 10.3390/children11060732 38929311 PMC11202073

[14] Ware SM Wilkinson JD Tariq M Schubert JA Sridhar A Colan SD et al. Genetic causes of cardiomyopathy in children: first results from the pediatric cardiomyopathy genes study. *J Am Heart Assoc.* (2021) 10:e017731. 10.1161/JAHA.120.017731 33906374 PMC8200745

[15] Wilkinson JD Landy DC Colan SD Towbin JA Sleeper LA Orav EJ et al. The pediatric cardiomyopathy registry and heart failure: key results from the first 15 years. *Heart Fail Clin.* (2010) 6:401–13. 10.1016/j.hfc.2010.05.002 20869642 PMC2946942

[16] Bogle C Colan SD Miyamoto SD Choudhry S Baez-Hernandez N Brickler MM et al. Treatment strategies for cardiomyopathy in children: a scientific statement from the American heart association. *Circulation.* (2023) 148:174–95. 10.1161/CIR.0000000000001151 37288568

